# Supplementary material for: Cervical Staphylococcus aureus Infection after Receiving the Third Dose of COVID-19 Vaccination: A Case Report
Source: Vaccines (Basel). 2022 Aug 8;10(8):1276. doi: 10.3390/vaccines10081276 (PMC9415974; doi:10.3390/vaccines10081276)
Supplement: Supplementary file 1 [file vaccines-10-01276-s001.zip › vaccines-1841367-supplementary.pdf]

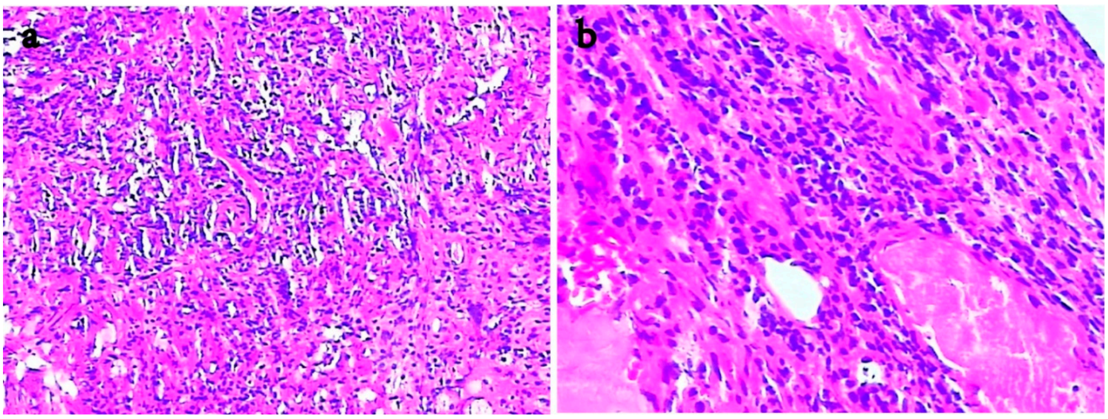

Supplementary Figure S1. Pathological results of the patient (a,b) The pathologic findings of the C5/6 disc showed extensive inflammatory exudation, suggesting chronic suppurative inflammation with dead bone formation. The typical Staphylococcus aureus is spherical, arranged in grape clusters under the microscope, and positive for Gram staining.
